# Intragenic Enhancers and Suppressors of Phytoene Desaturase Mutations in *Chlamydomonas reinhardtii*

**DOI:** 10.1371/journal.pone.0042196

**Published:** 2012-08-10

**Authors:** Phoi T. Tran, Marina N. Sharifi, Subhajit Poddar, Rachel M. Dent, Krishna K. Niyogi

**Affiliations:** Physical Biosciences Division, Lawrence Berkeley National Laboratory, Howard Hughes Medical Institute, Department of Plant and Microbial Biology, University of California, Berkeley, California, United States of America; United States Department of Agriculture, Agricultural Research Service, United States of America

## Abstract

Photosynthetic organisms synthesize carotenoids for harvesting light energy, photoprotection, and maintaining the structure and function of photosynthetic membranes. A light-sensitive, phytoene-accumulating mutant, *pds1-1*, was isolated in *Chlamydomonas reinhardtii* and found to be genetically linked to the phytoene desaturase (*PDS*) gene. PDS catalyzes the second step in carotenoid biosynthesis—the conversion of phytoene to ζ-carotene. Decreased accumulation of downstream colored carotenoids suggested that the *pds1-1* mutant is leaky for PDS activity. A screen for enhancers of the *pds1-1* mutation yielded the *pds1-2* allele, which completely lacks PDS activity. A second independent null mutant (*pds1-3*) was identified using DNA insertional mutagenesis. Both null mutants accumulate only phytoene and no other carotenoids. All three phytoene-accumulating mutants exhibited slower growth rates and reduced plating efficiency compared to wild-type cells and white phytoene synthase mutants. Insight into amino acid residues important for PDS activity was obtained through the characterization of intragenic suppressors of *pds1-2*. The suppressor mutants fell into three classes: revertants of the *pds1-1* point mutation, mutations that changed PDS amino acid residue Pro64 to Phe, and mutations that converted PDS residue Lys90 to Met. Characterization of *pds1-2* intragenic suppressors coupled with computational structure prediction of PDS suggest that amino acids at positions 90 and 143 are in close contact in the active PDS enzyme and have important roles in its structural stability and/or activity.

## Introduction

Carotenoids are a diverse class of isoprenoid pigments with important functions in nature. In plants and green algae they are C_40_ molecules with a long chain of conjugated double bonds that can absorb light energy and quench harmful molecules such as triplet chlorophylls and singlet oxygen [1], [2]. Plants synthesize carotenoids in chloroplasts for harvesting light energy, photoprotection, and maintaining the structure and function of photosynthetic membranes [1], [3], [4]. In photosynthetic tissues most carotenoids are bound to proteins localized in thylakoid membranes [5], [6], [7]. Besides their role in photosynthesis, carotenoids act as attractants for pollination and seed dispersal. In seeds, carotenoids help prevent seed aging and increase seed viability [8], [9]. Carotenoids can also be converted to the plant hormone, abscisic acid (ABA) [10], [11], [12], which promotes seed dormancy. Dietary carotenoids in animals have many functions as antioxidants, pigments, and precursors to vitamin A. A diet rich in carotenoids helps prevent eye diseases and can reduce the risk of cancers and UV damage to skin in humans [13], [14], [15].

**Figure 1:**
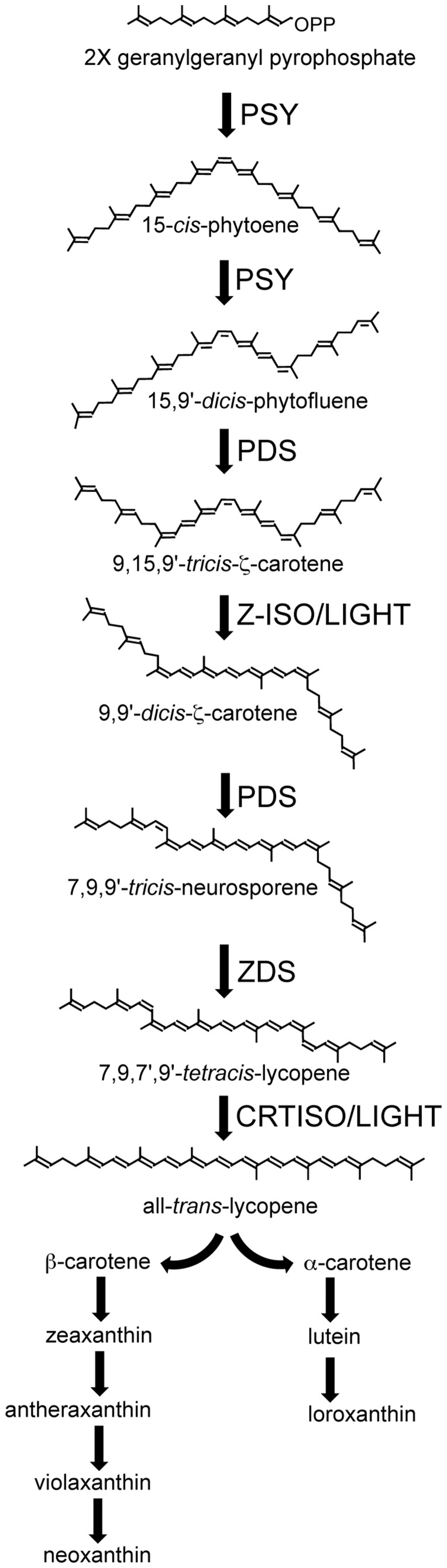
Carotenoid biosynthesis in plants and green algae. Geranylgeranyl pyrophosphate, phytoene, and phytofluene are all colorless compounds. Colored carotenoids include ζ-carotene and all carotenoids downstream. Xanthophylls include zeaxanthin, antheraxanthin, violaxanthin, neoxanthin, lutein, and loroxanthin (found in *C. reinhardtii*).

Carotenoid biosynthesis (Figure 1) involves four types of reactions: 1) condensation of two colorless geranylgeranylpyrophosphates (GGPP) molecules to form the colorless phytoene molecule, 2) desaturation and isomerization of phytoene to form red colored lycopene, 3) cyclization of lycopene to form beta-carotene and alpha-carotene and 4) addition of oxygen groups to form xanthophylls [16].

In plants and green algae, the first committed step of carotenoid biosynthesis is catalyzed by phytoene synthase (PSY), which joins two molecules of the colorless C_20_ compound geranylgeranyl diphosphate (GGPP) to form the colorless C_40_ carotene, 15-*cis*-phytoene. Two conjugated double bonds are then added to 15-*cis*-phytoene by phytoene desaturase (PDS), giving ζ-carotenes their characteristic light-yellow color. PDS catalyzes two successive dehydrogenation reactions, converting 15-*cis*-phytoene via the intermediate 15,9′-*dicis*-phytofluene to 9,15,9′-*tricis*-ζ-carotene. Plant and algal mutants affecting PSY and PDS activity accumulate GGPP and phytoene, respectively, resulting in albino seedlings for plants [17], [18], [19], [20], [21] and white-colored cells in algae [22].

*C. reinhardtii lts1* mutants impaired in PSY were previously characterized by McCarthy *et al.* [22], who isolated eleven “white” or carotenoid-less mutants, all of which were found to be affected in PSY activity and did not accumulate phytoene as would be expected for mutants with defects in PDS activity [22]. Vila *et al.* attempted to generate phytoene-accumulating mutants by post-transcriptional silencing of *PDS* expression through small interfering RNA (siRNA) and antisense RNA targeted to *PDS* [23]. Although they showed that *PDS* mRNA levels were reduced, carotenoid levels were unaffected, and phytoene did not accumulate [23]. Here we describe the successful isolation and characterization of *C. reinhardtii* mutants affecting *PDS* and offer an explanation as to why previous screens were unsuccessful.

## Materials and Methods

### Strains and growth conditions

The wild-type *C. reinhardtii* strains used in this work, 4A+ (*mt+*) and 4Ax5.2- (*mt−*), are in the 137c genetic background [24]. The polymorphic wild-type strain, S1D2 (*mt−*), was used in genetic linkage tests [25]. The *lts1-210* mutant has a null mutation in the *PSY* gene [22]. Cells were maintained on Tris-acetate-phosphate (TAP) agar medium [26] at 25°C in complete darkness. Unless otherwise specified, experiments were performed on cells grown in 50 ml of liquid TAP to a density of ∼5×10^6^ cells ml^−1^ in complete darkness with shaking at 120 rpm.

For norflurazon experiments, cells were spotted onto 35 ml of TAP-agar with norflurazon concentrations of 0.5 µM, 1 µM, 5 µM, 10 µM, 50 µM and 100 µM. Norflurazon was dissolved in methanol and diluted so that 100 µl were added per 35 ml of TAP-agar. TAP-only plates contained 100 µl of methanol. For pigment analysis, 4A+ cells were grown in 50 ml TAP plus 0 µM, 5 µM, or 10 µM norflurazon to a density of ∼5×10^6^ cells ml^−1^, and 4×10^7^ cells were harvested for high performance liquid chromatography (HPLC) analysis.

For light sensitivity assays, cells were inoculated into 150 µl of TAP in 96-well plates and grown for 2 days in the dark at 25°C. 5 µl of cells were then spotted onto TAP-agar and grown for 5 days in the dark. Cells were then shifted to 10 µMol photons m^−2^ sec^−1^ (vLL), 100 µMol photons m^−2^ sec^−1^ (LL), or 500 µMol photons m^−2^ sec^−1^ (HL) for 7 days. Dark-only cells were grown completely in the dark for 12 days. Cells were grown either in the dark or in LL for 2 weeks at 25°C prior to HPLC.

To determine plating efficiency, cells were grown to 2×10^6^ cells ml^−1^ and then counted using a hemacytometer. Since *pds1-3* cells tend to clump, all strains were incubated in 30 ml of water for 2 hours prior to cell counting allowing them to become single cells. The cells were then centrifuged at 3000× g for 5 min, and the resulting pellet was gently suspended in liquid TAP and plated onto TAP-agar plates using glass beads. The plates were incubated in the dark at 25°C for 2 weeks before colony forming units (CFU) were counted. Growth of white mutants compared to dark green wild-type cells was tested by mixing *lts1* or *pds1* cells in equal ratio to wild-type cells and plating onto TAP-agar. Plates were inoculated with 2500 cells for *pds1* strains and 1650 cells for wild-type and *lts1-210* strains and grown for 2 weeks in the dark.

To determine growth rates of 4A+, *lts1*, *pds1-3*, and *pds1-1*, 1×10^6^ cells were used to inoculate each of three 100 ml TAP cultures. The cultures were allowed to grow in the dark at 25°C with shaking at 120 rpm, and cells were counted every 12 hours for 1 week. Cell densities were measured with a Multisizer3 Coulter Counter (Beckman Coulter, Fullerton, CA).

### Mutagenesis

The *pds1-1*, *pds1-2*, P3-84, and *pds1-2* suppressor mutants were all generated using UV mutagenesis [22]. 4A+ cells were mutagenized to create *pds1-1* mutants, and *pds1-1* in turn, was mutagenized to generate the P3-84 strain. The *pds1-2* suppressor strains were generated by mutagenizing P3-84 cells. For each mutagenesis, 20 ml of cells (∼5×10^6^ cells ml^−1^) in an open 150 mm glass Petri dish were exposed to 90,000 µJ UV light cm^−2^. Cells were incubated overnight in the dark then plated onto TAP-agar with glass beads and further grown in the dark at 25°C until colonies became visible. For *pds1* enhancer mutants, light green, green brown, and white mutants were picked and further screened via HPLC for phytoene accumulation. To isolate suppressor mutants, P3-84 cells were UV mutagenized at 55,000 µJ UV light cm^−2^. A total of 35 TAP-agar plates were grown with 1.25×10^7^ mutagenized cells/plate. After plating onto TAP-agar, mutagenized cells were allowed to grow in the dark for 5 days followed by 2 weeks at a light intensity of 1 µMol photons m^−2^ sec^−1^. Green colonies were picked for further analysis.

The mutant *pds1-3* was generated by DNA insertional mutagenesis [24] using the pBC1 plasmid conferring paromomycin resistance. pBC1 was linearized with *Xba*I and 0.5 µg of the plasmid was used per transformation. Following transformation cells were resuspended in liquid TAP and placed in the dark with shaking at 120 rpm at 25°C to recover overnight. After recovery, mutagenized cells were centrifuged and resuspended in 300 µl TAP before being plated onto TAP-agar containing 10 µg ml^−1^ paromomycin. The cells were then kept in the dark at 25°C for 4 weeks to select for paromomycin-resistant colonies.

### HPLC analysis

Pigments were extracted and analyzed by HPLC from dark-grown liquid TAP cultures or from cells grown on TAP-agar as described previously [22]. Pigments were extracted from 1×10^8^ cells by vortexing in 200 µl of acetone for 30 seconds. After centrifugation at 20,000× g for 1 min, the supernatant was filtered through a 0.45-µM nylon filter and stored in the dark until HPLC analysis, when 25 µl of the pigment extract was separated on a reverse-phase C18 Spherisorb S5 ODS1 4.6-×250-mm cartridge column (Waters, Milford, MA) at 30°C. The carotenoids and chlorophylls were identified by their absorbance at 445 and 296 nm using a diode array detector. A standard curve of known concentrations of each purified compound was used for calculating chlorophyll and carotenoid concentrations. Since no commercially purified phytoene was available to create a standard curve, phytoene levels were compared using peak areas derived from HPLC analysis.

### Genetic analysis

All crosses were carried out according to Harris [26]. Because *pds1* mutants were extremely light sensitive, zygospores derived from *pds1* mutants were only exposed to 5 hours of vLL to induce germination. Germinated zygospores were dissected, and the resulting progeny were grown in complete darkness at 25°C on TAP-agar plates until colonies could be detected. The *pds1-1* (*mt+*), *pds1-3* (*mt+*), and P3-84 (*mt+*) strains were crossed to 4Ax5.2 (*mt−*). Progeny produced from crosses between 4Ax5.2 and *pds1-3* were tested for paromomycin resistance by growing the cells on TAP-agar plus 10 µg/ml paromomycin for 2 weeks in the dark.

For genetic linkage analysis, the *pds1-1* mutant was crossed to the S1D2 (*mt−*) strain. Genomic DNA was extracted from progeny resulting from this cross and used to amplify a 268-bp DNA fragment of the *PDS* gene with primers PDS4 (5′–ACCTTTCTGTTACACAAACCATGC-3′) and PDS7 (5′-TACACTGGTTTGGCACTCGTAGA-3′). The 268-bp PCR product was digested with *Scr*FI overnight before being run on a 3% Metaphor agarose gel (Cambrex, East Rutherford, NJ).

Vegetative diploids were generated by crossing *pds1-1* to an arginine-deficient strain with the *arg7-8* mutation [26], [27]. Progeny from this cross were maintained on TAP-agar supplemented with 50 µg/ml of L-arginine. The *pds1-1 arg7-8* (*mt−*) double mutants were selected by their light green color and their inability to grow on TAP-agar without arginine and then crossed to an arginine-deficient strain with the *arg7-1* allelic mutation. The mating mix was plated directly on TAP-agar without arginine plates and grown in LL at 25°C. After 10 days in the light, surviving colonies were picked and tested for their ploidy using mating-type PCR [27].

### DNA analysis

DNA and RNA were extracted from cells grown in liquid. For restriction enzyme site-directed amplification (RESDA)-PCR analysis, DNA was extracted from cells grown on TAP-agar plates for 14 days in the dark. DNA was extracted from cells as described previously [28], but without CsCl purification.

The *PDS* and *PSY* genes were sequenced from genomic DNA isolated from 4A+, *pds1-1*, P3-84, and *pds1-2* suppressor mutants. Sequencing primers were designed using Primer3 software [29] against the annotated *PDS* and *PSY* genes in the *C. reinhardtii* nuclear genome sequence from the Department of Energy Joint Genome Institute (JGI, http://genome.jgi-psf.org/Chlre4/Chlre4.home.html). PCR fragments were sequenced using the DYEnamic ET Terminator Cycle Sequencing kit (Amersham Biosciences, Piscataway, NJ) and then analyzed using an ABI 3100 automated DNA sequencer (Applied Biosystems, Foster City, CA). Primer pairs used to amplify and sequence regions carrying mutations in the *PDS* locus were: 1) C490019_17A (5′-GGACACCACCCAATCGTTCT-3′) and C490019_17B (5′-CTACAGCCGCCCTTACTGAC-3′) and 2) C490019_4A (5′-ATACGAACATATATACGTGGCACACT-3′) and C490019_4B (5′-ATGTTTAGCTCCTTGAAGACATTCAT-3′). Primers T-PSYF1 and PSYR2 [22] were used to amplify and sequence mutations in the *PSY* locus.

### RNA analysis

Total RNA for quantitative PCR (qPCR) and reverse-transcriptase (RT) PCR was prepared by first centrifuging cultures for 5 min at 3000 rpm followed by RNA extraction using 2 ml of Trizol reagent (Invitrogen, Carlsbad, CA) per 50 ml culture. Total RNA was resuspended in 30 µl DEPC-H_2_0 and treated with 1.5 µl RQ Rnase-free DNAse (Promega, Madison, WI) for 1 hour at 37°C, and RNA was purified from the reaction using RNAeasy columns (Qiagen, Valencia, CA).

First-strand cDNA was synthesized using Superscript Reverse Transcriptase III (Invitrogen, Carlsbad, CA). The first-strand synthesis reaction was set up with 1 µl of 50 µM oligo-dT_(20)_ primer, 1 µl 10 mM dNTPs, and 500 ng total RNA in a total volume of 13 µl. The cDNA synthesis reaction was incubated at 65°C for 5 minutes and quenched on ice for 1 minute before adding 4 µl 5× FS buffer, 1 µl 0.1 M DTT, 1 µl RnaseOUT, and 200 U enzyme. This was followed by a 50°C incubation for 1 hour, and finally 70°C for 15 min. 1 µl RNaseH was added to the reaction and incubated at 37°C for 20 min. 2 µl of the first-strand cDNA reaction was used as template for PCR amplification of specific transcripts with specific primers. Primers used for the amplification of tubulin as a positive control were tub-3 (5′-CGCCAAAGTACATCTCCATCC-3′) and tub-4 (5′-TAGGGGCTCTTCTTGGACA-3′) which produced a 285 bp fragment from genomic DNA and a 107 bp fragment from cDNA. Primers used to amplify the *PDS* transcript were PDSF_4 (5′-CTGCATGGAAGGATGAGGAT-3′) and MS069 (5′- TTGATCTCGGTGGGAAACA-3′).

For quantitative RT-PCR (qPCR), first-strand cDNA was synthesized from 1 µg total RNA with random primers (5′NNNNNNNNN) using Omniscript reverse transcriptase (Qiagen, Valencia, CA) according to the manufacturer's protocol. qPCR reactions were set up using 1 µl cDNA synthesis reaction diluted to 5 µl with sterile water as template, 2 µl of each primer at 2.5 µM concentration, and 10 µl 2× Sybr-green master mix (Qiagen, Valencia, CA) in a final volume of 20 µl. qPCR reactions were run on an ABI-7300 qPCR machine, with standard cycling. Transcript levels were quantified using the delta-delta Ct method. *CBLP* was used as the endogenous control gene, amplified with primers SWQ43 (5′-CAAGACCATCAAGCTGTGGA-3′) and SWQ44 (5′-ACACGATGATGGGGTTGGT-3′) which targeted the third exon. Primer pairs in the second exon of *PDS*, PDSF_4 (5′-CTGCATGGAAGGATGAGGAT-3′) and PDSR_4 (5′GAGTCGGGCATAGCAAAGAT-3′), and in the 3′ UTR, PDSF_3 (5′-ATCCGGAGGATTCAGGAGAC-3′) and PDSR_3 (5′-CAGAAGTCCGCACACTCAAA-3′), with approximately 150 bp amplicons were used for *PDS* expression analysis. Transcript levels were quantified using the delta-delta Ct method.

### Isolation and analysis of flanking genomic sequences

The insertion site of pBC1 in the DNA insertional mutant, *pds1-3*, was identified using RESDA-PCR [30]. A set of primary and secondary specific nested primers was designed to amplify genomic DNA flanking the vector insert. Flanking sequence was isolated with primary primer MS010 (5′-AATGCGGGCGTTGCAAGTCAAATC-3′) and secondary primer MS011A (5′-AATCTGCAAGCACGCTGCCTGATC-3′). Degenerate primers and the Q_0_ specific primer were those described in González-Ballester *et al.* [30], with the addition of a fifth degenerate primer constructed identically to the original four, replacing the original restriction enzyme cutting sites with the *StyI* site.

Two sequential PCR reactions were required to amplify the flanking sequence. The primary RESDA-PCR reaction was set up in a volume of 25 µl as follows: 5 pmol specific primary primer, 15 pmol degenerate primer, 2.5 µl Eppendorf 10× PCR Buffer Advanced, 2.5 µl 200 µM dNTPs, 0.3 µl Eppendorf Taq polymerase, and ∼80 ng genomic DNA template suspended in TE buffer. Primary reactions were diluted 1∶25 and used as template in secondary RESDA-PCR reactions which were set up in a volume of 25 µl as follows: 5 pmol specific secondary primer, 5 pmol Q_0_ specific primer, 2.5 µl Eppendorf 10× PCR Buffer Advanced, 2.5 µl 200, µM dNTPs, 0.3 µl Eppendorf Taq polymerase, and 1.5 µl diluted primary reaction. RESDA-PCR primary cycling parameters were as described in Dent *et al.* [24], whereas secondary cycling parameters were as described in González-Ballester *et al.* [30]. Secondary RESDA-PCR reactions were separated on 1% agarose gels, and reactions with amplification product(s) were purified for sequencing using either the Qiagen MinElute PCR purification kit (Qiagen, Valencia, CA), or the QIAquick gel extraction kit (Qiagen, Valencia, CA) for reactions that amplified multiple bands. 40–50 ng of the DNA obtained was sequenced with the plasmid specific primer RMD225 (5′-ATAAGCTTGATATCGAATTC-3′).

RESDA-PCR of *pds1-3* yielded *C. reinhardtii* genomic DNA flanking sequence that was used to design the primer MS039 (5′-GCCACGCCCTTGTAGTTGTA-3′) for further analysis of the insertion site. PCR with primer MS039 and RESDA-PCR secondary vector specific primers RMD225, RMD 271 (5′-CGAGCTCCCCGCTCGAGGTCGACG-3′), and MS011A (5′-AATCTGCAAGCACGCTGCCTGATC-3′) was performed. Primers were also designed within the *PDS* gene model at two locations upstream of the recovered flanking sequence: MS041A (5′-CTCCCTAACTCCCGCTCTTC-3′) and MS041B (5′-GTCCACGGTGGTCAGCTT-3′) were designed 500 bp upstream while MS031A (5′-GGTGGGTCATTTAGCACCTC-3′) and MS031B (5′-ATCCTCATCCTTCCATGCAG-3′) were designed 2.5 kb upstream.

### Bioinformatics and structural modeling

ChloroP 1.1 (http://www.cbs.dtu.dk/services/ChloroP/) was used to predict the presence and length of potential chloroplast transit peptides from translated protein sequences [31].

PDS protein sequences from *C. reinhardtii* (GenBank accession XP_001690859.1), *Synechocystis* sp. PCC 6803 (GenBank accession CAA44452.1) and *Arabidopsis thaliana* (GenBank accession AAA20109.1) were retrieved from NCBI protein database at http://www.ncbi.nlm.nih.gov/sites/entrez?db=protein [32]. *Ostreococcus tauri* PDS protein sequence was from the Joint Genome Institue (JGI, http://genome.jgi-psf.org/Ostta4/Ostta4.home.html, protein ID 21852). The protein sequences were aligned using ClustalW version 1.83 [33] at http://www.ch.embnet.org/software/ClustalW.html and shaded according to Blosum 62 matrix.

The *C. reinhardtii* PDS protein (GenBank accession XP_001690859.1) was submitted to 3DLigandSite web server (Ligand binding site prediction Server) at http://www.sbg.bio.ic.ac.uk/3dligandsite/ [34].

## Results

**Figure 2:**
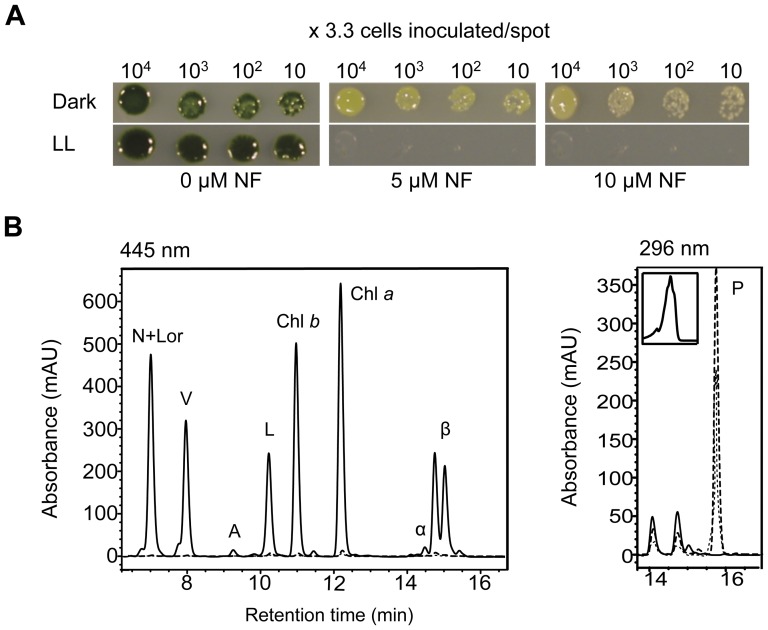
Phenotype of wild-type *C. reinhardtii* cells grown norflurazon. A). Growth of wild-type *C. reinhardtii* cells on 0, 5, and 10 µM norflurazon (NF) in LL (100 µMol photons m^−2^ sec^−1^) or in the dark. B). Overlay of HPLC results of carotenoid and chlorophyll pigments detected in dark-grown wild-type cells treated with 0 µM (solid lines), 5 µM (dashed lines), and 10 µM (dotted lines) NF. N+Lor (neoxanthin+loroxanthin); V (violaxanthin); A (antheraxanthin); L (Lutein); Chl *a* and Chl *b* (chlorophyll *a* and *b*); α-, ß- (α- and ß-carotenes); P (phytoene). Inset shows absorbance spectrum of phytoene peak at 296 nm.

*C. reinhardtii* wild-type cells (4A+) were grown on norflurazon to determine the expected phenotype of *pds1* mutants. Norflurazon is a bleaching herbicide that specifically inhibits PDS activity and therefore carotenoid biosynthesis [35], [36], [37]. When wild-type cells were grown on norflurazon, dark green cells became light green to almost white with increasing concentrations of norflurazon (Figure 2A). Cell growth was inhibited by norflurazon concentrations above 10 µM in the dark, whereas in low light cell growth was completely inhibited at 5 µM and higher. HPLC analysis of dark-grown cells showed that norflurazon-treated cells accumulated phytoene and had severe reductions in chlorophyll and carotenoids levels (Figure 2B). Phytoene was identified by its absorbance spectrum at 296 nm and its retention time (Figure 2B). Chlorophylls and other carotenoids were detected at 445 nm and also identified by their absorbance spectra and retention times (Figure 2B).

### A phytoene accumulating mutant: *pds1-1*

**Figure 3:**
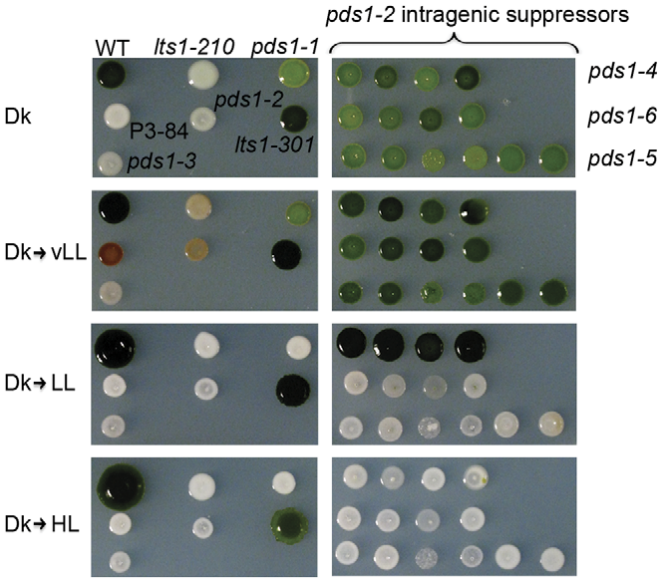
Light sensitivity of wild-type, *lts1* and *pds1* mutants. Cells were spotted onto TAP-agar and grown for 5 days in the dark before being exposed to light. All cells were grown for a total of 19 days. WT (wild-type), *lts1-210* (null *psy*), *pds1-1* (leaky *pds1*), P3-84 (*lts1-301 pds1-2*), *pds1-2*, *lts1-301* (leaky *psy*), and *pds1-3* (null *pds1*) are in the left column. In the right column are intragenic suppressors of *pds1-2* mutants (*pds1-4*, *pds1-5*, *pds1-6*), all in the *lts1-301* genetic background. Light intensities: Dk (dark), vLL (10 µMol photons m^−2^ sec^−1^), LL (100 µMol photons m^−2^ sec^−1^), HL (500 µMol photons m^−2^ sec^−1^).

**Figure 4:**
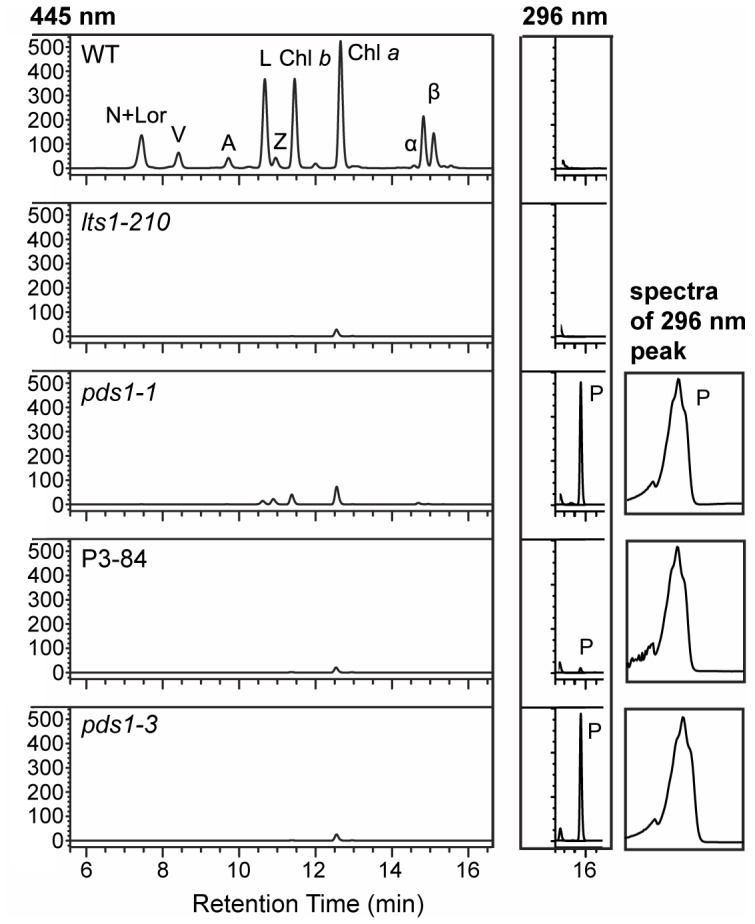
Chlorophyll and carotenoid profiles of PDS-activity deficient mutants. Chlorophylls and carotenoids were detected at 445 nm and phytoene was detected at 296 nm. Absorbance spectra are shown for the 296 nm phytoene peak present in both *pds1-1* and *pds1-3* mutants and small peak detected in P3-84. Pigments were extracted from a total of 1×10^8^ cells for each sample and analyzed via HPLC coupled with a diode array detector. N+Lor (neoxanthin+loroxanthin); V (violaxanthin); A (antheraxanthin); L (lutein); Z (zeaxanthin); Chl *a* and Chl *b* (chlorophyll *a* and *b*); α-, ß- (α- and ß-carotenes); P (phytoene).

Based on the results of norflurazon inhibition, *C. reinhardtii* mutants that are defective in PDS activity were predicted to have a light to very pale green color. From a UV mutagenesis screen, 135 light green, pale green, white, and green/brown color mutants were picked and analyzed by HPLC for pigment abnormalities. The *pds1-1* mutant was identified from this screen—it was light green and accumulated phytoene (Figure 3 and 4). However, *pds1-1* still produced carotenoids downstream of phytoene including ß-carotene, lutein, antheraxanthin, violaxanthin, and neoxanthin (Figure 4) at ∼5% the levels found in wild-type cells (Table 1). The *pds1-1* mutant accumulated 5-fold more chlorophyll than *lts1* mutants but only ∼12% the level detected in wild-type cells. The chlorophyll to colored carotenoid ratio for wild-type cells was 3.2∶1, whereas in *pds1-1* the ratio was 8.7∶1. Wild-type and *lts1* mutants did not accumulate any phytoene, whereas *pds1-1* mutants accumulated significant levels of phytoene (Figure 4).

**Table 1:** Quantification of chlorophyll and carotenoid content of dark-grown *lts1* and *pds1* mutants.

	total chl (fmol/cell)	chl *a/b* ratio	total colored carotenoids (fmol/cell)	total xanthophylls (fmol/cell)	lutein (fmol/cell)	zeaxanthin (fmol/cell)	phytoene (total peak area)
**Wild-type**	0.5201±0.0404	2.17±0.23	0.1616±0.0307	0.1079±0.0208	0.0377±0.0291	0.0069±0.0030	0
***lts1-301*** ** (leaky ** ***psy*** **)**	0.2297±0.0243	2.24±0.51	0.0391±0.0056	0.0306±0.0051	0.0111±0.0032	0.0099±0.0016	0
***lts1-210*** ** (null ** ***psy*** **)**	0.0128±0.0041	39.24±1.09	0	0	0	0	0
***pds1-1*** ** (leaky ** ***pds*** **)**	0.0655±0.0076	2.67±0.15	0.0075±0.0006	0.0061±0.0006	0.0025±0.0006	0.0031±0.0	3,605±177
***pds1-2*** ** (null ** ***pds*** **)**	0.0113±0.0020	13.84±0.27	0	0	0	0	2,642±232
***pds1-3*** ** (null ** ***pds*** **)**	0.0177±0.0084	13.84±0.27	0	0	0	0	4,048±907
***lts1-301 pds1-2***	0.0096±0.0031	14.44±0.42	0	0	0	0	167±34
**suppressor ** ***pds1-4***	0.1726±0.0643	2.08±0.12	0.0313±0.0107	0.0247±0.0076	0.0088±0.0023	0.0059±0.0019	0
**suppressor ** ***pds1-5***	0.2464±0.0917	2.12±0.32	0.0363±0.0075	0.0283±0.0055	0.0102±0.0022	0.0070±0.0029	24±11
**suppressor ** ***pds1-6***	0.2297±0.0744	2.32±0.27	0.0298±0.0072	0.0236±0.0074	0.0088±0.0022	0.0066±0.0029	8±0.68

Chlorophyll (Chl) and carotenoid quantities (represented as fmol/cell) were extracted from a total of 1×10^8^ cells for each sample with 200 µl of acetone. Colored carotenoids detected in *C. reinhardtii* include α-carotene, β-carotene, lutein, violaxanthin, antheraxanthin, neoxanthin, loroxanthin, zeaxanthin. Total xanthophylls include lutein, loroxanthin, violaxanthin, antheraxanthin, neoxanthin, and zeaxanthin. Averages and standard deviations are from three independent cultures.

Similar to *lts1* mutants, the *pds1-1* mutant was found to be very light sensitive. After growth in the dark for four days, *pds1-1* died after being exposed to more than 24 hours of vLL (Figure 3). In vLL cells died and turned brown, whereas at higher light intensities (LL and HL) cells bleached completely and turned white (Figure 3). In contrast, wild-type cells grew well at all light intensities including HL.

Genetic analysis of *pds1-1* revealed that the *pds1* phenotype is caused by a single, recessive nuclear mutation. Crosses between *pds1-1* and wild-type cells produced tetrads that segregated 2∶2 for the *pds1-1* mutant phenotype (light colored, phytoene accumulation, and reduced levels of colored carotenoids) and the wild-type phenotype (dark green, no phytoene, and normal levels of carotenoids) (Table 2). Dominance testing using heterozygous *pds1-1/PDS1* vegetative diploids showed the *pds1-1* mutation is recessive.

**Table 2:** Tetrad analysis of *pds1-1* and *pds1-3* crossed to wild-type.

	PD∶NPD (complete tetrads)	Total progeny	WT progeny	Mutant progeny	Mutant progeny recombinant for paromomycin marker
***pds1-1*** ** (** ***mt+*** **)×WT (** ***mt−*** **)**	10∶0	106	54	52	N/A
***pds1-3*** ** (** ***mt+*** **)×WT (** ***mt−*** **)**	7∶0	145	88	57	0

WT = wild type, PD = parental ditype, NPD = non-parental ditype.

**Figure 5:**
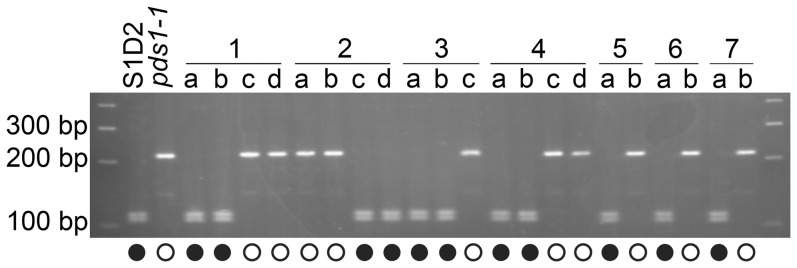
The *PDS* gene is genetically linked to the *pds1-1* mutant phenotype. A marker located within the *PDS* locus cosegregated with the light green, phytoene-accumulating mutant phenotype of *pds1-1*. Amplification and *Scr*FI digestion of a 268 bp fragment of the *PDS* gene containing a single nucleotide polymorphism in exon 2 was used to score progeny from crosses between *pds1-1* and a polymorphic wild-type strain (S1D2). Seven full and partial tetrads were scored: individual progeny within tetrads are labeled “a, b, c, d”. Solid circles indicate dark green progeny with wild-type carotenoid composition while open circles indicate light green progeny with phytoene accumulation.

The *pds1-1* mutant was crossed to the polymorphic wild-type strain S1D2 in order to map the mutation relative to the annotated *PDS* gene. A total of 21 progeny were isolated from this cross: 12 from complete tetrads and 9 from incomplete tetrads. A marker for the *PDS* locus on chromosome 12 amplified a 268 bp PCR product from both *pds1-1* and S1D2. Digestion of the 268 bp PCR product with *Scr*FI yielded 215 and 52 bp fragments from *pds1-1*, whereas 111, 104, 26, and 25 bp fragments were produced from S1D2. DNA fragments smaller than 100 bp could not be visualized. When the *PDS* marker was tested on DNA isolated from the progeny, the light green phenotype cosegregated with the polymorphism found in *pds1-1* (215 and 52 bp), while dark green progeny yielded fragments similar to S1D2 (111, 104, 26, and 25 bp) (Figure 5). This result shows that the light green phenotype is linked to the *PDS* locus and that a mutation in *PDS* is likely to be responsible for the light green color and phytoene accumulation in *pds1-1*.

**Figure 6:**
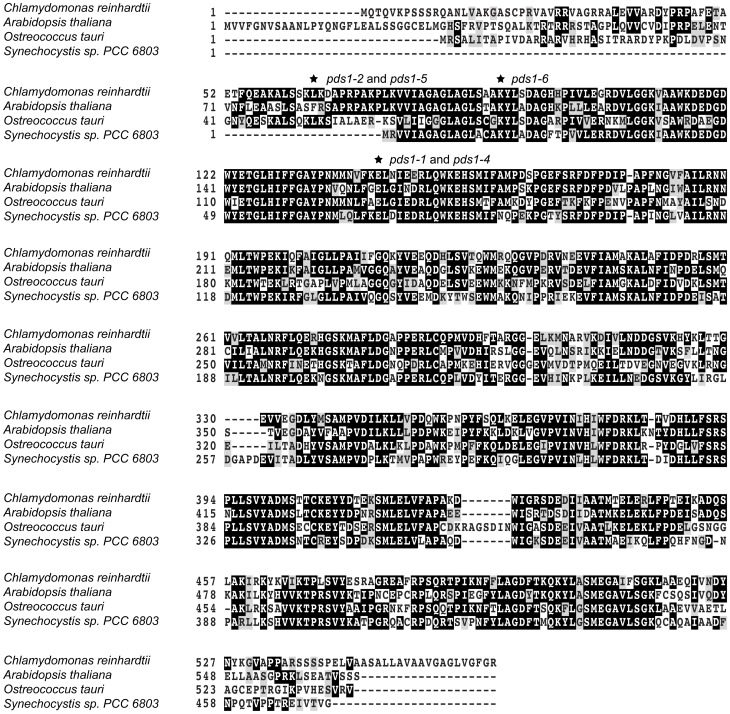
Multiple sequence alignment of PDS protein sequences. Alignment of PDS amino acid sequences from eukaryotic green algae, *Chlamydomonas* and *Ostreococcus*; a plant, *Arabidopsis*; and a cyanobacterium, *Synechocystis sp.* PCC6803. Conserved residues were scored using Blosum 62 matrix, with darker shading indicating higher conservation and no shading low conservation. Asterisks mark positions of mutations in *pds1* alleles.

The *PDS* locus was sequenced from *pds1-1* to discover if a mutation in this locus was responsible for the phytoene-accumulating, light green phenotype. The *Chlamydomonas* nuclear genome sequence of *PDS* is 4030 bp and the predicted protein is 564 amino acids long. Amplification and sequencing of the *PDS* locus identified a single base pair change in exon two of *pds1-1*. The point mutation consisted of a G/C to A/T transition, resulting in an E143K missense change in deduced PDS protein sequence (Figure 6). A multiple sequence alignment of predicted PDS protein sequences from wild-type *C. reinhardtii*, *O. tauri*, *Synechocystis* sp PCC6803, and *A.thaliana* and revealed that the amino acid change occurred in the conserved dinucleotide (FAD)-dependent oxidoreductase/amine oxidase domain of the PDS protein (Figure 6).

### Isolation of *pds1-2* as an intragenic enhancer of *pds1-1*

**Figure 7:**
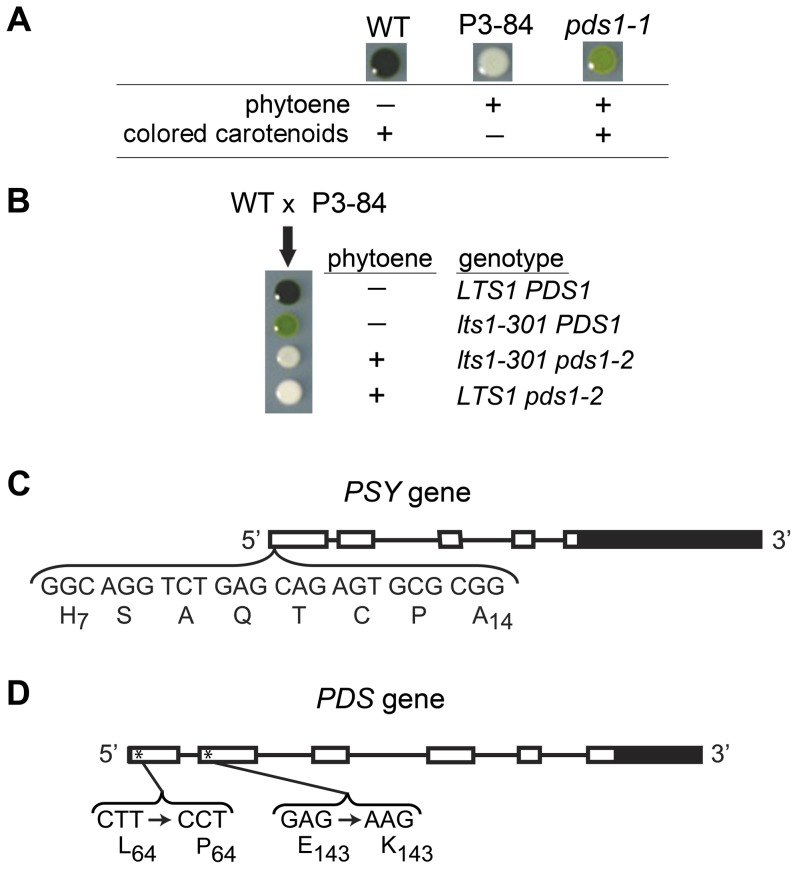
Analysis of enhancer strain P3-84 (*lts1-301 pds1-2*) and intragenic suppressors of *pds1-2* mutants. A). Color and pigment phenotype of dark-grown wild type (WT), P3-84, and *pds1-1* mutants. (−) indicates no accumulation while (+) indicates presence of phytoene or colored carotenoids. Colored carotenoids include all carotenoids downstream of phytofluene. B). Tetratype tetrad phenotype from crosses between wild-type and P3-84 cells. The presence (+) or absence (−) of phytoene is indicated for each progeny along with their corresponding genotypes. C). Structure of the *PSY* gene in *C. reinhardtii*. UTRs are indicated by solid boxes, exons by open boxes and four introns by lines. The bracket highlights the eight amino acids and their corresponding nucleotides deleted from the putative chloroplast transit peptide in *lts1-301*, P3-84, and in *pds1-2* suppressor mutants. Subscript numbers note position of the amino acid residue in the wild-type PSY protein. D). Structure of the *PDS* gene in *C. reinhardtii*. UTRs are indicated by solid boxes, exons by open boxes and five introns by lines. The brackets highlight the two missense mutations found in P3-84. Subscript numbers note position of the amino acid residue in the wild-type PDS protein. Asterisks mark approximate location of mutations.

Because *pds1-1* still synthesizes colored carotenoids, a second round of UV mutagenesis was conducted to find enhancer mutants that eliminated PDS activity. Light green *pds1-1* cells were mutagenized, and a white mutant, P3-84, was isolated (Figure 3 and Figure 7A) that had a similar pigment profile as null *psy* (*lts1-210*) mutants, except that P3-84 accumulated a low level of phytoene (Figure 4). When P3-84 was crossed to wild-type cells, the tetratype tetrad progeny gave unexpected pigment phenotypes: two white progeny with phytoene accumulation and no other carotenoids, one dark green mutant with wild-type carotenoid composition and levels, and one light green mutant with lower carotenoid levels (Figure 7B). The original light green, phytoene-accumulating *pds1-1* pigment phenotype was not recovered. To determine if the P3-84 mutant phenotype was due to mutations in either the *PSY* or *PDS* gene, both genes were sequenced. Sequencing results revealed new mutations in both *PSY* and *PDS* genes in P3-84. An in-frame deletion of 24 bp removed eight amino acid residues from positions 7 to 14 (H_7_SAQTCPA_14_) in the putative chloroplast transit peptide of PSY (Figure 7C). This new allele of *PSY* was named *lts1-301*. The *PDS* locus was found to carry two point mutations: the original *pds1-1* mutation (E143K) and an additional T to C transition resulting in the conversion of a leucine residue at position 64 to a proline residue (L64P) (Figure 7D, Table 3). This double mutant allele of *PDS* was named *pds1-2*.

**Table 3:** Summary of mutants described in this work.

Strain(s)	Genotype	PSY mutation	PDS mutation
*lts1-210*	*lts1-210*	W60stop	n/a
*lts1-301*	*lts1-301*	in-frame deletion of PSY residues 7–14 (HSAQTCPA)	n/a
SP60.90	*pds1-1*	n/a	E143K
*pds1-2*	*pds1-2*	n/a	L64P E143K
T29-3	*pds1-3*	n/a	pBC1 insertion
P3-84	*lts1-301 pds1-2*	in-frame deletion of PSY residues 7–14 (HSAQTCPA	L64P E143K
csp6,10,14,15	*lts1-301 pds1-4*	in-frame deletion of PSY residues 7–14 (HSAQTCPA	L64P
csp3,4,5,9,11,16,17	*lts1-301 pds1-5*	in-frame deletion of PSY residues 7–14 (HSAQTCPA	L64F E143K
csp1,7,8,12,13	*lts1-301 pds1-6*	in-frame deletion of PSY residues 7–14 (HSAQTCPA	L64P K90M E143K

The *PDS* and *PSY* sequencing results from P3-84 explained the unexpected tetratype phenotypes recovered in the cross between P3-84 and wild-type. The two parental phenotypes were represented: dark green wild-type and white P3-84 (Figure 7A and 7B). For the two unexpected phenotypes, the light-green, no phytoene accumulating phenotype belonged to progeny with reduced PSY activity (Figure 7B). Sequencing of *PSY* and *PDS* genes from this progeny (*lts1-301*) revealed that it has the eight amino acid chloroplast transit peptide deletion in *PSY* and no mutations in *PDS* (Figure 7C). The *lts1-301* strain synthesizes wild-type carotenoids, but at reduced levels, indicating that PSY function is reduced or “leaky” (Table 1) possibly because of inefficient transport of the PSY protein into the chloroplast. The second unexpected phenotype, white plus phytoene accumulation, belonged to progeny with wild-type *PSY* and the two mutations in *PDS* (L64P and E143K) (Figure 7B). This second white progeny, *pds1-2*, is an intragenic enhancer mutant for *pds1-1* since the only carotenoid detected was phytoene (Table 1). Both P3-84 (*lts1-301 pds1-2*) and *pds1-2* survive only in the dark. Similar to *lts1-210* and *pds1-1* mutants, they die when cultured under very low light. In contrast, *lts1-301* is very light tolerant, growing almost as well as wild-type cells in HL (Figure 3).

### *pds1-3* is a null allele derived from DNA insertional mutagenesis

An additional white, phytoene-accumulating mutant, *pds1-3*, was isolated from a DNA insertional mutagenesis screen based on its sensitivity to light and white color. Similar to *pds1-1* mutants, *pds1-3* bleached and died at vLL intensities (Figure 3), and it also accumulated phytoene (Figure 4). Unlike *pds1-1*, however, it does not synthesize any colored carotenoids (Table 1).

Tetrads from crosses with wild-type segregated 2∶2 for the *pds1-3* and wild-type phenotypes (Table 2), indicating that the *pds1-3* mutant phenotype is controlled by a single gene. Co-segregation of the mutant phenotype with paromomycin resistance also indicated that the mutation is tagged by the transforming plasmid (Table 2).

**Figure 8:**
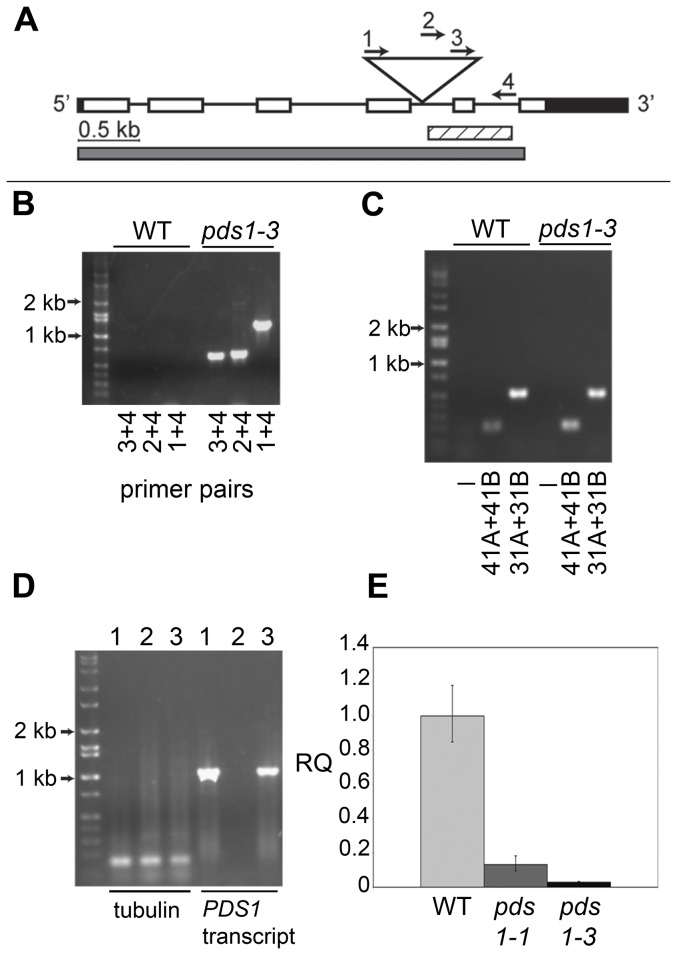
Analysis of *pds1*-3 DNA insertional mutant. A). Schematic of the *C. reinhardtii PDS* gene showing DNA insertion location (triangle), region amplified in flanking sequence tag (striped bar), and region of transcript not detectable by RT-PCR in *pds1-3* (gray bar). UTRs are indicated by black bars and exons by open bars. Genomic DNA spanning the 5′ UTR to the 4^th^ exon could be amplified by PCR in *pds1-3*. B). No amplification in wild-type (WT) and amplification in *pds1-3* with three vector-specific primers (1, 2, 3) and one primer in *PDS* genomic DNA (4), indicated by arrows in panel A, confirming insert location in *pds1-3*. C). Successful amplification of genomic DNA and DNA sequencing of *PDS* on the opposite side of insertion from flanking sequence tag in wild-type and *pds1-3*. Amplification products were obtained from genomic DNA 2.5 kb (primers MS031A and MS031B, in exon 1) and 500 bp (primers MS041A and MS041B, in exon 4) distant from insertion site. D). Amplification of *PDS* transcript (gray bar in panel A) from total RNA in wild-type (1), *pds1-3* (2), and *pds1-1* (3), with the amplification of tubulin as a positive control. E). Relative *PDS* transcript levels in wild-type cells (light gray bar), *pds1-1* (dark gray bar ), and *pds1-3* (black bar). Relative quantification (RQ) fold-change values to the calibrator (WT *PDS* transcript levels) are shown.

To identify the mutation responsible for the white, phytoene-accumulating phenotype of the *pds1-3* mutant, RESDA-PCR was used to recover a flanking sequence tag for one end of the vector insert in *pds1-3*. The flanking sequence was used as a query in a BLAST search for homologous sequences [38] against the Department of Energy (DOE) Joint Genome Institute (JGI) *Chlamydomonas reinhardtii* v4 genome (www.jgi.doe.gov/chlamy) and found to have significant identity to a 331 bp sequence on Chromosome 12. Flanking sequence analysis indicated that the insertion interrupts an intron in *PDS* (Figure 8A). A primer was designed within the *Chlamydomonas* genomic DNA flanking the putative insert location obtained from RESDA-PCR for *pds1-3*, and PCR with this primer and three nested primers within the vector was performed (Figure 8A). Successful amplification confirmed the location of the plasmid vector in *pds1-3* genomic DNA (Figure 8B). Recovery of flanking sequence at the other end of the insert was unsuccessful, however. One reason may be because the insertion of foreign DNA into *Chlamydomonas* genomic DNA is often accompanied by a deletion [24]. To determine whether a significant deletion was present in *pds1-3*, PCR primers were designed within the *PDS* genomic DNA on the side of the insertion for which no flanking sequence could be recovered. MS031A and MS031B primers amplified a 498 bp product from *pds1-3* genomic DNA, 500 bp downstream from the insertion point, and primers MS041A and MS041B amplified a 200 bp fragment 2.5 kb distant from the site of insertion. Successful amplification and DNA sequencing of these PCR products indicated that a large deletion did not accompany the plasmid insertion (Figure 8C).

The *PDS* transcript is present in *pds1-1* (Figure 8D), although at a reduced level: ∼13% of the level found in wild-type (Figure 8E). In contrast, no *PDS* transcript was detectable by RT-PCR or qPCR in *pds1-3* (Figures 8D and 8E).

### Growth defects of *pds1* mutants

**Figure 9:**
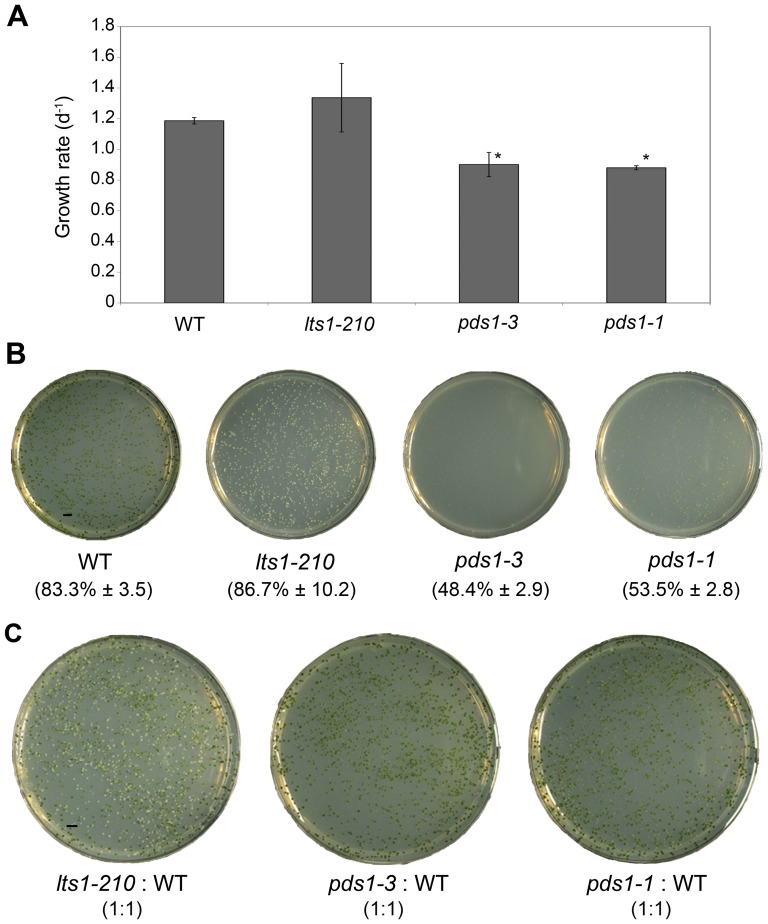
Results of plating assays of wild-type, *lts1-210*, *pds1-3*, and *pds1-1*. A). Growth rate per day for wild-type, *lts1-210* (null *psy*), *pds1-3*, and *pds1-1*. Biological triplicates of each strain were grown in the dark in liquid TAP on a shaker. *Significantly differently from *lts1-210* and wild-type values under the same conditions using a two-tailed t test (P<0.05). B). Percent of cells that survived plating with glass beads (% survival ± standard deviation) after 12 days of growth in the dark. Scale bar represents 5 mm. C). ∼1∶1 ratio of carotenoid mutant to wild-type cells after 12 days of growth in the dark. After accounting for plating efficiencies, the expected CFU/plate was 1250 CFU/plate for *pds1* and 1320 CFU/plate for wild-type and *lts1-210* strains. Scale bar represents 5 mm.

Multiple independent alleles of *lts1* but no *pds1* mutants were isolated in a previous screen for white mutants of *C. reinhardtii* [22]. To understand why *pds1* mutants were not found, the growth rates of *pds1* mutants were compared to *lts1-210* and wild-type cells. Comparison of growth rates in liquid TAP medium in the dark showed that *pds1-1* and *pds1-3* mutants grew more slowly than either *lts1-210* or wild-type cells (Figure 9A).

Differences in growth between *pds1* mutants and white *lts1-210* or wild-type cells were more pronounced in plating assays. First, the plating efficiency of *pds1*, *lts1*, and wild-type strains was measured as colony-forming units (CFU). After 3 weeks growth in the dark, the plating efficiency of *pds1-1* was calculated as 53.5%±2.8; *pds1-3* was 48.4%±2.9; *lts1-210* was 86.7%±10.2; and wild type was 83.3%±3.5 (Figure 9B).

A second plating experiment was performed to assess how visible *pds1* mutants are in a background of wild-type cells when grown in a ∼1∶1 ratio (Figure 9C). After accounting for the ∼50% and ∼80% observed plating efficiencies for *pds1* strains and wild-type/*lts1-210* strains, respectively, the expected CFU was 1250 CFU/plate for *pds1* mutants and 1320 CFU/plate for wild-type and *lts1-210* strains. On TAP-agar plates with a 1∶1 ratio of *lts1-210* to wild-type cells, white *lts1-210* colonies were easily identified; they were as densely populated and equal in diameter to wild-type colonies (Figure 9C). In contrast, it was difficult to identify light green *pds1-1* and white *pds1-3* mutants among wild-type colonies because their colonies were frequently half the diameter or smaller than wild-type colonies and fewer in number (Figure 9C). Of the carotenoid mutants tested, *pds1-3* colonies were the smallest and the least dense. Because of their small size, it was difficult to determine the color of some *pds1* colonies and as a result, they could have been mistaken for extremely small wild-type colonies or not been detected at all in a screen for white mutants [22].

### Intragenic *pds1-2* suppressor mutants

To gain further insight into amino acid residues important for PDS structure and function, mutations that suppressed the white phenotype of *pds1-2* were isolated. Sixteen light green *pds1-2* suppressor mutants falling into three allelic classes were isolated from UV mutagenesis of the white P3-84 strain (*lts1-301 pds1-2*).

**Figure 10:**
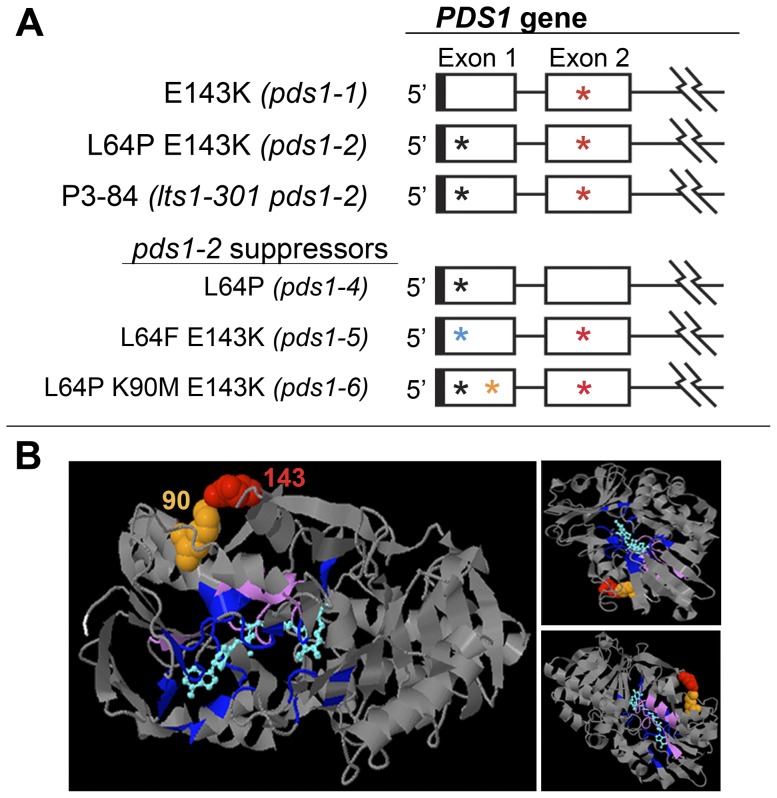
Analysis of intragenic suppressors of *pds1-2* mutants. A). Schematic depiction of *pds1-1*, *P3-84*, *pds1-2*, and *pds1-2* suppressors (*pds1-4*, *pds1-5*, and *pds1-6*). Cartoon of the *C. reinhardtii PDS* gene showing only exons one and two. Jagged lines indicate a partial depiction of the *PDS* gene. UTRs are indicated by solid boxes, exons by open boxes and introns by lines. Asterisks mark positions of mutations in *pds1-1*, *pds1-2*, P3-84 and *pds1-2* suppressor mutants. Black and blue asterisks represent mutations L64P and L64F, respectively. Orange asterisk in exon 1 signifies location of the K90M mutation, and red asterisks in exon 2 signify the E143K mutation. B). 3DLigandSite structural prediction of the *C. reinhardtii* PDS protein showing positions of amino acid residues mutated in *pds1-1* and *pds1-6* mutants, E143K and K90M, respectively. L64P and L64F were not mapped because the first 71 amino acids of the N-terminus had no structural prediction. Ligand and wild-type amino acids corresponding to mutated residues were colored as follows: position 90 (spacefilling, orange); position 143 (spacefilling, red); ligand [NAD(P)/FAD] (cyan); predicted ligand binding sites (indigo); start of predicted N-terminus (amino acid residue 72, white); and carotenoid binding site proposed by Armstrong *et al.* (amino acid residues 492–517, lavender). Three different perspectives of the predicted structure are shown.

*PSY* and *PDS* genes were both sequenced from the *pds1-2* suppressor mutants to identify any revertants and/or additional mutations. All 16 suppressor mutants retained the chloroplast transit peptide mutation in the *PSY* gene (*lts1-301*) from strain P3-84 (Figure 7C). Four of these strains, csp6, csp10, csp14, and csp15, had a reversion of the *pds1-1* mutation: a transition from “A/T” (Lys143) back to wild-type “G/C” (Glu143) (Figures 6 and 10A; Table 3). These suppressors retained the L64P mutation, which was named *pds1-4*. The second class of intragenic *pds1-2* suppressor mutants, *pds1-5*, had the original *pds1-1* mutation (E143K) plus a new *pds1* mutation, which converted the *pds1-2* mutation (L64P) in exon one to L64F (Figure 7, 10A). Seven *pds1-5* strains were isolated: csp3, csp4, csp5, csp9, csp11, csp16 and csp17 (Figure 3, csp17 not shown; Table 3). The third class of intragenic *pds1-2* suppressor mutants had three mutations in *PDS*: E143K from *pds1-1*, L64P from *pds1-2* and a new mutation, K90M. The methionine at position 90 resulted from a transversion mutation that changed the wild-type “A” to a “T (Figures 6 and 10A). In this third allelic class, *pds1-6*, five strains were isolated: csp1, csp7, csp8, csp12, and csp13 (Figure 3, csp8 not shown; Table 3).

Light green intragenic suppressor mutants of *pds1-2* were more light tolerant than light green *pds1-1* and white *pds1* mutants but less light tolerant than medium green *lts1-301* single mutants (Figure 3). All suppressor mutants grew well under vLL, but only *pds1-4* mutants could survive in LL. The *pds1-5* and *pds1-6* mutants bleached and died in LL (Figure 3). No suppressor mutant survived in HL. Although the suppressor mutants were less light tolerant than *lts1-301* single mutants, pigment analysis of *pds1-2* suppressor mutants showed that all three classes synthesize the full spectrum of wild-type colored carotenoids, but at ∼20% of the levels found in wild-type cells, similar to *lts1-301* (Table 1). Comparison of total xanthophylls, known for their photoprotective properties [39], [40], did not reveal any significant differences between suppressor mutants and *lts1-301*. Zeaxanthin, a xanthophyll particularly important for photoprotection [40], [41], [42], was elevated 1.6 fold in *lts1-301* compared to suppressor mutants and 1.4 fold higher than in wild-type cells. The levels of lutein were significantly lower than in wild-type cells but not significantly different among suppressor mutants or *lts1-301* mutants. Two of the three *pds1-2* suppressor mutant classes still accumulated phytoene. The *pds1-5* and *pds1-6* strains accumulated phytoene at ∼17% and ∼6%, respectively, of the levels present in the starting strain P3-84 (Table 1). Like wild-type cells, *pds1-4* mutants did not accumulate any phytoene (Table 1).

### PDS structural prediction

The 3DLigandSite web server predicted that the *C. reinhardtii* PDS protein has a structure most similar to a human monoamine oxidase, C2c70B. Both C2c70B and the *C. reinhardtii* PDS proteins were classified as oxidoreductases and had 12% identity to each other. 3DLigandSite predicted the presence of a dinucleotide-binding motif [NAD(P) or FAD] in the center of the PDS protein (cyan blue, Figure 10B) and potential ligand binding sites (indigo, Figure 10B) [43], [44]. The C-terminus of bacterial carotenoid dehydrogenases was proposed to contain a hydrophobic carotenoid-binding pocket [46], which was also found conserved among cyanobacteria, algae, and plants [45], [46]. In *C. reinhardtii* this region spans amino acid residues 492–517 (Figure 10B, lavender).

In the predicted PDS protein structure, amino acid residue 143 (mutated in both *pds1-1* and in *pds1-2* suppressor mutants) and amino acid residue 90 (mutated in *pds1-6*), were adjacent to one another, and in spacefilling mode, in physical contact (Figure 10B, Glu143, red and Lys90, orange). In the wild-type PDS protein these amino acids are Glu143 and Lys90. Amino acid residue 64, which is affected in *pds1-2* and the suppressor mutants could not be visualized, because no structure was predicted for the first 71 amino acid residues of the N-terminus (or the last 22 residues of the C-terminus) of *C. reinhardtii* PDS.

## Discussion

Metabolic pathways are commonly regulated at their early steps in order to conserve resources and control accumulation of possibly harmful intermediates. Studies in other organisms indicate that the first two steps of carotenoid biosynthesis, catalyzed by PSY and PDS, are likely points of regulation for the whole pathway. *PSY* was reported to be regulated in tomato [47], pepper [48], mustard [49], [50], corn [51], [52], [53], sunflower [54] and algae [55], [56] by light and/or carotenoid content. *PDS* was found to be regulated or rate-limiting in algae [56], [57], [58], [59], potato [60], pepper [48], and tomato [46], [47].

Previous studies have identified many *C. reinhardtii lts1* mutants affecting *PSY*, but no *C. reinhardtii pds1* mutants had been isolated until this study. A phytoene-accumulating *C. reinhardtii* mutant was previously reported in a study by Stolbova [61], who observed that the light-sensitive *lts4* mutant accumulated phytoene, but without any significant change in pigment composition [61]. The *lts4* mutation was mapped to chromosome 11, indicating that it was not a *pds1* mutant since the only copy of the *C. reinhardtii PDS* gene is on chromosome 12 [62]. The *lts4* mutation might instead be linked to a plastid terminal oxidase (PTOX) or to plastoquinone biosynthesis, both of which are necessary for phytoene desaturation [19], [63], [64].

Similar to *lts1* mutants, *pds1* mutants are extremely light sensitive and pale in color. Mutants lacking PSY (*lts1-210*) or PDS activity (*pds1-2* and *pds1-3*) accumulate no colored carotenoids (Figure 4) and die when exposed to even very low light intensities (Figure 3). Surprisingly, the leaky *pds1-1* mutant was as light sensitive as *lts1* and *pds1* null mutants even though it was able to accumulate colored carotenoids. The light sensitive phenotype of *pds1-1* indicates that the amount of colored carotenoids present in *pds1-1* provides insufficient protection from even very low light.

### The deleterious effect of phytoene accumulation in *C. reinhardtii*

The major difference between the white *lts1* and *pds1* mutants is the accumulation of phytoene in *pds1*. Phytoene-accumulating *pds1-1* and *pds1-3* mutants grew more slowly in complete darkness and had a lower plating efficiency than wild-type or *lts1-210* cells (Figure 9). Together, these phenotypes could explain the difficulty in isolating *pds1* mutants in previous screens [22].

Light sensitivity assays of *pds1-2* suppressor mutants also suggest that phytoene accumulation has a deleterious effect on the fitness of *C. reinhardtii* cells. All three classes of suppressor mutants accumulated significant amounts of colored carotenoids, but the *pds1-5* and *pds1-6* mutants that accumulated low levels of phytoene were more light sensitive than *pds1-4* mutants that did not (Figure 3). The *pds1-4* mutants did not have more photoprotective carotenoids than either *pds1-5* and *pds1-6* mutants (Table 1), so their ability to survive in higher light intensities cannot be attributed to the presence of higher levels of total colored carotenoids or to a specific photoprotective carotenoid such as lutein or zeaxanthin.

A harmful effect of phytoene accumulation might explain the occurrence of the *lts1-301* mutation in the *pds1-1* enhancer strain, P3-84 (*lts1-301 pds1-2*). The mutation in *PSY* might have arisen secondarily, enhancing the growth and survival of cells with loss of PDS function by mitigating the accumulation of phytoene. The *lts1-301* mutation decreases the flux of metabolites entering carotenoid biosynthesis and therefore reduces the amount of phytoene that accumulates in the cell (Figure 4).

In *pds* mutants of plants, the possible effects of phytoene accumulation are difficult to assess. Generally, studies have shown that impairment of PDS activity result in phytoene accumulation and pleiotropic defects in plants. *Arabidopsis* [18], [19], [65], maize [20], [66], rice [17], [66], [67], and tobacco plants [21] with impaired PDS activity accumulate phytoene, are lethal at the seedling stage, have stunted growth, exhibit albinism, and in the case of maize and rice, seeds experience vivipary. Light-exposed, norflurazon-treated plants also accumulate phytoene and produce albino seedlings or white leaf sectors [36], [47], [68], [69]. However, these morphological defects are not exclusive to plants with reduced PDS activity and phytoene accumulation. Mutants in other steps of the carotenoid pathway and in metabolic pathways that feed substrates directly into carotenoid biosynthesis also produce mutants with albinism, vivipary, and stunted growth. These mutants include *psy* mutants [21], *zds* mutants [17], [70], GGPP synthase mutants [71] and mutants of the plastidic methylerythritol 4-phosphate (MEP) pathway [72]. Carotenoid and abscisic acid deficiency is probably the primary cause of the adverse phenotypes in plant *pds* mutants.

### Insight into the structure of the *C. reinhardtii* PDS protein

Amino acid residues affected in *pds1-1*, *pds1-2*, and intragenic suppressors of *pds1-2* mutants must play an important role in PDS structure and/or function. Phyre prediction of the PDS protein structure placed amino acid residues affected in *pds1-1* and *pds1-6* mutants in close proximity with one another. In wild-type PDS, these amino acids are negatively charged Glu143 and positively charged Lys90, respectively. 3DLigandSite did not identify them as residues required for FAD/NAD(P) binding. Because of their predicted physical proximity to each other, it is possible that these residues form an ion pair that is important for proper folding of PDS. In *pds1-1*, Glu143(−) is converted to Lys143(+). This would result in two positively charged residues, Lys143(+) and Lys90(+), in direct contact, which would presumably introduce electrostatic repulsion and possibly promote protein destabilization. Electrostatic repulsion might be alleviated in *pds1-6*, which substitutes Lys90(+) with an uncharged methionine. The *pds1-6* mutants carrying this amino acid change were light green in color, not dark green like wild-type cells, indicating that full PDS activity was not recovered. Changes to amino acid residues 90 and 143 result in less PDS activity, but further investigation is required to determine if this is due to decreases in PDS enzyme specific activity or PDS protein accumulation. The reason for lower PDS activity in the six mutant alleles of *pds1* could be addressed by immunoblot analysis with a specific anti-PDS antibody.

P3-84, *pds1-2*, and all intragenic suppressor mutants have mutations that affect amino acid residue 64, but no structure was predicted for the first 71 amino acid residues of the PDS N-terminus. In wild-type cells, this residue is hydrophobic leucine, whereas in P3-84 and *pds1-2* this residue was converted to cyclic proline, which in conjunction with the *pds1-1* mutation severely impaired PDS activity. The replacement of proline with a different hydrophobic amino acid, phenylalanine, allowed *pds1-5* suppressor mutants to partially recover PDS activity. The first 70–80 residues of the N-terminus of PDS is not present in the cyanobacterium *Synechocystis* indicating that this region is not critical for PDS activity (Figure 6). Instead, it may be involved in chloroplast targeting or perhaps insertion into the thylakoid membrane. Several studies using protein blotting and immunogold labeling have localized PDS proteins in the thylakoid membranes [20], [73].

In summary, we have isolated and characterized six alleles of *pds1* in *C. reinhardtii*. Comparisons of *lts1* and *pds1* mutants suggest that phytoene accumulation is deleterious and that PDS may be an important control point in understanding and engineering carotenoid biosynthesis. Homology modeling and structural analysis of the *pds1* mutations have also provided insight into the PDS protein structure and function.
